# Data-Driven Coping Phenotypes in Schizophrenia: Clinical and Functional Correlates from The Italian Network for Research on Psychoses study

**DOI:** 10.1192/j.eurpsy.2026.12139

**Published:** 2026-07-31

**Authors:** P. Pezzella, E. Caporusso, A. Perrottelli, L. Giuliani, A. Melillo, F. F. Marzocchi, M. Amore, P. Rocca, A. Rossi, A. Bertolino, G. M. Giordano, A. Mucci, S. Galderisi, M. Maj

**Affiliations:** 1 Department of Psychiatry, University of Campania “Luigi Vanvitelli”, Naples; 2 Department of Neurosciences, Rehabilitation, Ophthalmology, Genetics and Maternal and Child Health, Section of Psychiatry, University of Genoa, Genoa; 3 Department of Neuroscience, Section of Psychiatry, University of Turin, Turin; 4 Department of Biotechnological and Applied Clinical Sciences, Section of Psychiatry, University of L’Aquila, L’Aquila; 5 Department of Basic Medical Science, Neuroscience and Sense Organs, University of Bari ‘Aldo Moro’, Bari, Italy

## Abstract

**Introduction:**

Coping strategies are key determinants of clinical and functional outcomes in schizophrenia, with maladaptive patterns consistently linked to more severe psychopathology, impaired social functioning, and unfavorable illness trajectories. However, the heterogeneity of coping strategies in this population remains poorly understood, limiting the possibility of developing targeted psychosocial interventions.

**Objectives:**

This study aimed to identify distinct coping profiles in individuals with schizophrenia and to explore their clinical, cognitive, and functional correlates.

**Methods:**

Within the Italian Network for Research on Psychoses study, 861 clinically stable, community-dwelling patients with schizophrenia were recruited through 24 university psychiatric clinics and mental health departments. Participants were comprehensively assessed on psychopathology, premorbid functioning, real-life functioning, neurocognition and social cognition, and coping strategies (using the COPE-BRIEF scale). The 14 COPE domains were standardized (z-scores) and entered into a k-means clustering analysis, which identified four distinct coping profiles.

**Results:**

These profiles differed significantly across clinical and functional variables. Premorbid academic and social functioning varied between groups (χ²(3)=32.6 and χ²(3)=19.5, both p<.001), with the most impaired scores in the hyper-engaged (cluster 3) and disengaged/avoidant (cluster 4) groups. Real-life functioning also differed (work χ²(3)=31.1, interpersonal χ²(3)=41.1, everyday life activities χ²(3)=35.5; all p<.001), with clusters 3 and 4 showing poorer outcomes compared to the more balanced (cluster 1) and mixed-intense (cluster 2) groups. Psychopathology was significantly different, with higher negative (avolition χ²(3)=41.7, expressive deficits χ²(3)=29.2), positive (χ²(3)=27.5) and disorganized symptoms (χ²(3)=42.1; all p<.001) in clusters 3 and 4. Neurocognition (χ²(3)=42.4, p<.001) and social cognition (χ²(3)=28.2, p<.001) were also more compromised in these two groups. Substance abuse prevalence was (χ²(3)=8.5, p=0.037) higher in clusters 2 and 3.

**Conclusions:**

These findings highlight clinically meaningful coping phenotypes, suggesting that extreme strategies, either hyperengagement or disengagement, are linked to greater functional impairment and symptom severity, underscoring the need for tailored psychosocial interventions.

**Disclosure of Interest:**

None Declared

